# Lung ultrasound for the diagnosis of subpleural consolidations - a review of the veterinary and human literature

**DOI:** 10.1186/s13028-024-00784-4

**Published:** 2024-11-29

**Authors:** Michał Gajewski

**Affiliations:** Vetcardia Veterinary Clinic, 11 Kijowska Street, Warsaw, 03-743 Poland

**Keywords:** Atelectasis, Cats, Consolidation, Dogs, Lung ultrasound, LUS, Neoplasia, Pneumonia

## Abstract

Lung ultrasound (LUS) is an imaging modality of growing importance in human medicine. LUS has been extensively applied to human patients. Guidelines have been created for internal medicine, describing ultrasonographic features of various lung pathologic processes. Such guidelines do not exist for veterinary medicine, and studies on the utility of LUS in companion animals are limited. Therefore, this review compares conclusions from veterinary studies to recommendations in human medicine for the detection of subpleural consolidations beyond the application of LUS as a point-of-care modality in emergency and critical care.

## Background

Lung ultrasound (LUS) examination is a relatively new yet rapidly evolving diagnostic modality for detecting the most common pathologies in the lungs. LUS was introduced in human medicine in the 1980s and 1990s. The development of the BLUE protocol by Daniel Lichtenstein [1] was one of the turning points for implementing the technique in emergency and critical care. LUS proved highly useful and was frequently performed since the beginning of the COVID-19 pandemic in 2020. As a result, much scientific data was collected from which LUS recommendations were created. This imaging modality has been thoroughly studied in human medicine and is an essential tool for increasing diagnostic accuracy in dyspneic patients [2]. The sensitivity and specificity of LUS are comparable to computed tomography (CT) of the chest and exceed that of chest radiography (CXR) [2]. In veterinary medicine, however, LUS has not been studied as extensively, especially for its application in small animal internal medicine. Since LUS examination in veterinary patients is primarily utilized in emergencies (as a point-of-care ultrasound, POCUS), many studies focus on assessing B-line artifacts as a method of diagnosing cardiogenic pulmonary edema or differentiating causes of dyspnea in acute disease states [3–5]. However, the literature on pulmonary consolidations and their relevance in veterinary patients is scarce. Therefore, this literature review is focused on this critical yet undervalued aspect of LUS. The decision to focus on lung pathologies associated with consolidations was dictated by the need to highlight the paucity of literature data and to compare the available studies with the recommendations from human medicine.

Of the whole range of studies on LUS in small animals, there are only a few describing the utility of LUS for the diagnosis of parenchymal lung diseases (i.e., pneumonia, interstitial lung diseases, atelectasis, and malignancies) associated with lung consolidations in small animal patients [6–16]. Unfortunately, there are discrepancies in methodology, terminology, and interpretation of LUS in small animals, which adds to the confusion of terms, lowering the applicability of this diagnostic tool. It is worth noting that LUS will play a different role depending on the clinical context in which it is used. In emergency cases requiring quick intervention, it is justified to use a simplified diagnostic scheme to guide therapeutic interventions quickly. A different approach should be considered for stable internal medicine patients, in which time allows for a more detailed LUS approach. This universality of using LUS is an advantage of this diagnostic modality. Still, it may also be the source of some inaccuracies and differences in terminology and interpretation regarding subpleural consolidations. This review aims to outline the diagnostic criteria for various lung consolidations in humans and compare them with veterinary publications regarding subpleural consolidations. Currently, veterinary LUS is mainly perceived as a screening test that needs more clarity among veterinary radiologists regarding its place in diagnostic workups and the use of adjunct techniques such as color Doppler (CD) and power Doppler (PD) analysis. This review will guide interest in ways to approach respiratory patients with LUS, describe ultrasound modalities recommended in people to increase its diagnostic potential and point out potential future developments in applying LUS in various clinical settings.

### Search strategy

A literature review was performed by searching the scientific databases (PubMed, Internet Archive Scholar, and ScienceDirect) for keywords “lung ultrasound,” “dogs,” “cats,” “small animals,” “consolidation,” “pneumonia,” “neoplasm,” “dyspnea,” “dyspneic,” and “lung consolidation.” No specific publication period was set; however, studies included in this review were published between 2017 and 2023. A total of 29 studies were found, out of which 12 focused on analyzing lung consolidations. Studies that assessed vertical artifacts caused by cardiogenic pulmonary edema were excluded. The recommendations from human internal medicine were used as a reference for the critical analysis of the veterinary literature [2, 17].

## Review

### LUS in human medicine – recommendations for internal medicine

LUS plays a vital role in human medicine, finding application in many fields, from emergency and intensive care to cardiology, internal medicine, pediatrics, and neonatology. For this modality to be utilized effectively and consistently, there needs to be a general understanding and consensus on the technique and interpretation of acquired images. Otherwise, differences in the interpretation of artifacts and subpleural consolidations can lead to incorrect conclusions. Two published guidelines and recommendations will be presented in this review and serve as a reference for the veterinary literature review. These recent publications were created as consensus statements per the modified Delphi voting method in two or three rounds. The construction of these two reference papers is different; therefore, each will be presented separately. However, three main thoughts common for both guidelines are: (1) LUS should cover the largest possible area of the lung surface and be performed according to a protocol tailored to the clinical condition of the patient (stable vs. unstable); (2) trained clinicians should perform LUS, and training programs in LUS should be a part of all specialty programs and students’ curriculum; and (3) ideally LUS should be performed with both a convex and linear probe.

### New international guidelines and consensus on the use of lung ultrasound

These guidelines [17] were published in 2023 and are presented as a list of 20 statements (classified as technical, clinical, educational, and safety), each describing the current state of LUS, its recommendations, and future development [17]. Reviewing all these statements in this article would be impractical; however, the author wishes to present a few key messages on the technical and clinical aspects of LUS included in these recommendations.

### Technical statements

Regarding technical aspects, the recommendations highlight the need to standardize imaging protocols, including the MI (mechanical index) range, probe and scanner type, imaging frequency, and focal and imaging depth. These factors have a high impact on acquired images, both in terms of non-artifactual (e.g., pleural line, consolidations, pleural effusion) and artifactual (vertical and horizontal artifacts) features [17]. These guidelines provide further information on various ultrasound machine settings and suggestions for uniformity to make the LUS examination more reproducible.

The guidelines also suggest the need for a consensus on LUS terminology describing artifacts and abnormalities, with a common dictionary with correlative LUS examples. Including such statements in the guidelines indicates concern about discrepancies in terminology, even among specialists in human medicine.

### Clinical statements

The guidelines cover many clinical statements regarding LUS examination, like the importance of covering the largest possible area of the lung surface, while acknowledging that in emergency medicine, the LUS approach should be more rapid and limited. In general, however, the accuracy of the examination increases with the surface area covered [17]. A distinction may be made in LUS formats for rapid point-of-care examination in critical and unstable patients versus a more complete, comprehensive, and time-consuming LUS examination whenever clinical conditions allow it in stable patients.

The guidelines include two statements (no. 11 and 17) specifically focused on the utility of LUS in assessing subpleural consolidations and pneumonia. The first states that LUS, used alone or along other imaging techniques, has high accuracy in recognizing subpleural consolidations, but the authors didn’t give a specific number demonstrating that accuracy. These may be divided into inflammatory lesions, atelectasis, infarction, and metastases. Subpleural consolidations should be examined with the combined use of convex and linear probes to differentiate inflammatory, infarctual, neoplastic, or atelectatic lesions [17]. Only consolidations in direct contact with visceral pleura may be detected in LUS. The guidelines also suggest using contrast-enhanced LUS (CEUS) whenever possible to obtain further information on subpleural consolidations. CEUS allows the differentiation of vascularity from the pulmonary or bronchial artery, and the extent of enhancement may differ in necrotic, inflammatory, and neoplastic lesions. However, further studies are needed to better define the use of CEUS for subpleural lesion characterization [17]. CEUS is rarely performed in veterinary medicine, mainly because it is a challenging procedure requiring advanced training and is often cost-prohibitive. The veterinary studies on the utility of CEUS in lung pathologies are described in the veterinary section of this manuscript.

Finally, the guidelines state that the most common LUS features of pneumonia are consolidation with irregular margins, air bronchograms, and air trapping sign; B-lines; and the presence of pleural effusion and irregularity of the pleural line. The most important feature of the consolidation, apart from its size and margins, is the presence of air bronchograms. Dynamic air bronchograms, along with patient history and clinical profile, are essential for ruling out obstructive atelectasis. Fluid bronchograms may be differentiated from blood vessels using Doppler imaging. A suggested glossary of LUS terms and their explanation is presented in Table 1.

**Table 1:** Glossary of terms used in LUS

Term	Explanation
Consolidation with an irregular margin	An airless hypoechoic portion of lung with a serrated hyperechoic lower margin; analogous to the “shred sign” in Vet BLUE^®^.
Dynamic air bronchogram	Hyperechoic foci creating a branching pattern within the consolidation and representing air within the airways; the echoes appear in greater numbers at inspiration and partially disappear in expiration, a dynamic phenomenon created by the movement of air within the airways.
Air trapping	Presence of small, isolated hyperechoic foci within the bronchial tree that are too few to form a bronchogram. Represent small pockets of air trapped within the bronchial tree.
Static air bronchogram	Hyperechoic foci creating a branching pattern within the consolidation and representing air within the airways; they don’t change with respirations and remain visible in both inspiration and expiration.
Fluid bronchogram	Hypoechoic, long, fluid-filled spaces within the consolidation representing effusion within the bronchial tree.
Normal vascular pattern	A branching, tree-like pattern created by blood flow within a consolidation, visible in CD or PD mode; the vessels course along the bronchial tree.
Chaotic vascular pattern	Presence of a distorted, random vascular pattern within the consolidation, visible in CD or PD mode; blood flow may be continuous (neovascularization) or pulsatile.
B-lines	Hyperechoic vertical artifacts that originate from the pleural line and extend to the bottom end of the screen and move in synchrony with respirations; may or may not obliterate A-lines.
Pleural effusion	Presence of anechoic, hypoechoic, or echoic fluid in the pleural cavity; fluid separates the lung from the thoracic wall, thereby causing the disappearance of the pleural line.
Homogenous consolidation	An airless fragment of the lung that has a uniform echogenicity and echostructure; represents the “tissue sign” in Vet BLUE^®^.
Wedge-shaped consolidation	A small, airless area of subpleural consolidation resembling a wedge in a normal, aerated lung; represents the “wedge-sign” in Vet BLUE^®^.
Heterogenous consolidation	An airless fragment of the lung that has variable echogenicity and echostructure within its borders.
Vascular sign (flow amputation)	A small fragment of a vessel with a visible blood flow in CD or PD mode that terminates abruptly at the proximal edge (i.e. at the narrow apex located lower on the screen) of a wedge-shaped consolidation.
Local fluid directly above the lesion	A small portion of free fluid within the pleural cavity present only within the borders of a subpleural consolidation.
Local interstitial lesions	Small fragment of pleural line abnormalities (thickening, blurring, irregularity, discontinuity) with or without vertical artifacts of various length originating from the pleural line.
Infiltration of adjacent structures	Extension of the pathologic process from the lung through anatomical borders of the lung into the pleural cavity or thoracic wall structures.
Additional vascularity from intercostal vessels	Presence of blood flow within the branches originating from intercostal vessels in the thoracic wall, extending into the subpleural consolidation in CD or PD mode.

LUS, lung ultrasound; CD, color Doppler; PD, power Doppler

### Recommendations for lung ultrasound in internal medicine in people

A working group of Polish experts published the recommendations for LUS in internal medicine (POLLUS-IM) in 2018 [18]. These recommendations were updated in 2020 due to the emergence of many new studies and meta-analyses that provided additional input to the previous version [2]. The process of updating the recommendations was divided into several stages: literature review, assessment of literature data quality, and expert evaluation of that data in three rounds. The recommendations were also categorized according to their strength. These updated POLLUS-IM recommendations [2] consist of statements describing specific pathological states, supported by experts’ additional suggestions and tips on practical aspects of LUS examination. The recommendations are categorized according to the pathology: pneumothorax, pleural fluid, lung pathologies associated with pulmonary interstitial lesions, pulmonary pathologies associated with consolidations, the diaphragm, and other indications [2].

### Lung pathologies associated with consolidations

Pulmonary pathologies associated with consolidations comprise a heterogenic group of disease states. The POLLUS-IM recommendations describe a consolidation as a subpleural hypoechoic area with a liver-like structure [2]. This description is short but broad enough to encompass all essential lung pathologies. The authors of the recommendations denote that the etiology of subpleural consolidations is varied, and most commonly, they result from pneumonia, atelectasis, malignant processes (both primary and metastatic), pulmonary embolism, and lung contusion. They also state that LUS may be superior to chest radiography (CXR) for confirming the presence of lung consolidations, and it is an accurate and quick diagnostic tool for differentiating the causes of acute dyspnea, including pneumonia, acute heart failure, and exacerbation of chronic obstructive pulmonary disease [2]. However, the reference examination for assessing pulmonary lesions is computed tomography (CT), performed according to a protocol suitable for an initial diagnosis. The authors also point out that the coexistence of more than one pathology of the respiratory system is common, so LUS findings may represent different pathologies in one patient [2].

#### Pneumonia

Pulmonary lesions associated with pneumonia of bacterial origin are as follows: consolidation with an irregular marginal contour, air bronchogram, the air trapping sign, B-lines, normal vascular pattern in CD and PD options, and the presence of pleural effusion [2]. The experts’ commentary on that statement divides the features of an inflammatory lung lesion into three categories: parenchymal (consolidation with an irregular margin, dynamic air bronchogram, and/or air trapping sign), vascular (normal blood flow as demonstrated in CD or PD), and pleural (pleural effusion). The sonographic features of lung pathologies associated with consolidation and their explanation are presented in Tables 2 and 1. The recommendations emphasize that LUS is superior to CXR for confirming the presence of pneumonia, and clinically suspected pneumonia with typical inflammatory lesions in LUS does not require a confirmation with CXR [2]. The POLLUS-IM recommendations state that the diagnostic sensitivity of LUS for diagnosing pneumonia amounts to 87–95%, and specificity to 80–96%, and is comparable to CT in diagnosing community-acquired pneumonia. The interpretation of the LUS examination should include the entire patient profile to place in the proper context.

**Table 2:** Summary of sonographic features of various subpleural consolidations according to the POLLUS-IM recommendations

Type of lesion	Features
Pneumonia	Parenchymal: consolidation with an irregular margin; dynamic air bronchogram and/or air trapping
	Vascular: normal vascular pattern
	Pleural: B-lines, pleural effusion
Compression atelectasis	Pleural effusion; homogenous consolidation; static air bronchogram; air trapping; normal vascular pattern
Resorption atelectasis	Homogenous consolidation; fluid bronchogram; static air bronchogram; normal vascular pattern; potential visualization of a pathological mass central to the consolidation (i.e. central to the atelectatic lung peripherally)
Pulmonary embolism	Consolidation - wedge-shaped or oval/rounded; centrally located echo; vascular sign (or flow amputation); local pleural fluid directly peripheral to the lesion; local interstitial lesions
Pulmonary neoplasia	Infiltration of adjacent structures; heterogeneous sonomorphology; chaotic vascular pattern; concomitant resorption atelectasis and/or fluid; additional vascularity originating from intercostal vessels

POLLUS-IM, Polish recommendations for lung ultrasound in internal medicine; B-lines, vertical artifacts arising from the pleural line, reaching the bottom of the screen, and moving in synchrony with lung sliding

#### Atelectasis

Atelectasis is divided into two subcategories: compression atelectasis (i.e., caused by external pressure, mainly from pleural effusion) or resorption atelectasis (e.g., from airway obstruction). Table 2 presents the sonographic features of each type of atelectasis.

#### Pulmonary embolism

Pulmonary embolism has the following LUS features: consolidation, mostly wedge-shaped or oval/rounded, centrally located echo, flow amputation in the CD option (the so-called vascular sign), local fluid directly above the subpleural lesion, and local interstitial lesions (see Table 2). The lack of lesions typical of pulmonary embolism does not exclude the diagnosis. Pulmonary embolism may be suspected if one typical abnormality is detected in LUS and can be diagnosed if ≥ 2 typical lesions are detected [2].

#### Neoplasia

Neoplastic lesions located beneath the pleural line have the following features on LUS: infiltration of adjacent structures, heterogeneous sonomorphology of the consolidation, chaotic vascular pattern in CD and PD options, and concomitant resorption atelectasis and/or fluid (see Table 2) [2, 19–21]. The lesions suspected of having a malignant etiology (whether primary or metastatic) may be biopsied under ultrasound guidance, which is a common diagnostic approach. The recommendations also indicate the possibility of observing additional vascularity originating from intercostal vessels within the subpleural malignant lesions on CD or PD.

### Veterinary literature review

The recommendations from human medicine may serve as guidance for veterinary studies in the process of developing veterinary-specific techniques of LUS examination. To verify if such guidance is needed, one must first challenge the available literature to contrast these recommendations with data presented in veterinary studies. When reviewing veterinary literature, it is necessary to note that most of these studies were published before or at the same time as the guidelines for human medicine (Table 3). Therefore, critical remarks should be read as suggested recommendations rather than a general dismissal of the study’s relevance.

**Table 3:** Studies on subpleural consolidations in veterinary medicine included in the review

Authors	Title	Publication date	Scanning technique
Dicker et al.	Diagnosis of pulmonary contusions with point-of-care lung ultrasonography and thoracic radiography compared to thoracic computed tomography in dogs with motor vehicle trauma: 29 cases (2017–2018).	21 October 2020	Vet BLUE^®^
Armenise et al.	Veterinary-focused assessment with sonography for trauma-airway, breathing, circulation, disability and exposure: a prospective observational study in 64 canine trauma patients.	13 December 2018	VetFAST-ABCDE
Ward et al.	Lung ultrasonography findings in dogs with various underlying causes of cough.	01 September 2019	Vet BLUE^®^
Lam et al.	Influence of concurrent lower respiratory tract disease on point-of-care lung ultrasound in small-breed dogs with myxomatous mitral valve disease.	27 April 2022	Vet BLUE*
Lin et al.	Usefulness of chest ultrasonography in predicting diagnosis in non-emergency small animal patients with lung parenchymal and pleural disease.	18 December 2020	Unspecified
Cole et al.	Diagnostic accuracy of a lung ultrasound protocol (Vet BLUE) for detection of pleural fluid, pneumothorax and lung pathology in dogs and cats.	26 January 2021	Vet BLUE*
Pacholec et al.	Lung ultrasound nodule sign for detection of pulmonary nodule lesions in dogs: Comparison to thoracic radiography using computed tomography as the criterion standard.	31 July 2021	Vet BLUE^®^
Łobaczewski et al.	Integrated basic heart and lung ultrasound examination for the differentiation between bacterial pneumonia and lung neoplasm in fogs-a new diagnostic algorithm.	29 April 2022	Vet BLUE*
Łobaczewski et al.	Lung ultrasound for imaging of B-lines in dogs and cats-a prospective study investigating agreement between three types of transducers and the accuracy in diagnosing cardiogenic pulmonary edema, pneumonia and lung neoplasia.	16 November 2021	Vet BLUE*
Fernandes Rodrigues et al.	Comparison of lung ultrasound, chest radiographs, C-reactive protein, and clinical findings in dogs treated for aspiration pneumonia.	5 March 2022	Modified VetFAST-ABCDE
Citi et al.	Thoracic ultrasound: a method for the work-up in dogs and cats with acute dyspnea.	2017	Unspecified
Kraszewska et al.	Case report: application of color Doppler sonography for the assessment of pulmonary consolidations in a dog.	8 December 2023	VetLus
Tindale et al.	Clinical characteristics and long-term outcome of lung lobe torsions in cats: a review of 10 cases (2000–2021).	1 November 2021	Unspecified
Caivano et al.	Contrast-enhanced ultrasonographic findings in three dogs with lung lobe torsion.	24 October 2015	CEUS
Belmudes et al.	Lung lobe torsion in 15 dogs: peripheral band sign on ultrasound.	18 January 2021	Unspecified
Rick et al.	Contrast-enhanced ultrasonography characteristics of intrathoracic mass lesions in 36 dogs and 24 cats.	26 November 2018	CEUS
Linta et al.	The feasibility of contrast enhanced ultrasonography (CEUS) in the diagnosis of non-cardiac thoracic disorders of dogs and cats.	25 May 2017	CEUS

VetFAST-ABCDE = veterinary focused assessment with sonography for trauma-airway, breathing, circulation, disability and exposure; Vet BLUE^®^ = veterinary brief lung ultrasound exam, performed by a trained operator; Vet BLUE* = veterinary brief lung ultrasound exam, performed by an operator not trained by the originator of the Vet BLUE^®^; VetLus = veterinary lung ultrasound; CEUS = contrast-enhanced ultrasound

One of the most widely recognized veterinary LUS protocols is the Vet BLUE, designed by Lisciandro as a regional, pattern-based approach similar to how lung radiography is interpreted. Its views include the caudodorsal, perihilar, middle, and cranial lung regions. The format is used not only for emergency and critical care but also as an extension of the physical exam for respiratory patients or suspects, i.e., cardiogenic pulmonary edema, dogs with cough, and lung metastasis. Vet BLUE-related clinical studies have focused on the detection of B-lines, their scoring using a numerical system (0,1,2,3,>3 and infinity), scoring systems by combining scores at regional views, and their distribution, in addition to detecting subpleural consolidations, i.e., shred sign, tissue sign, nodule sign, and wedge sign. It is known from research on healthy dogs and cats that during Vet BLUE B-lines are uncommon [3]. The shred sign, a Lichtenstein term, has irregular borders and hyperechoic foci representing air bronchograms [3]. The publication distinguishes static and dynamic air bronchograms and points out that static air bronchograms represent atelectasis, and dynamic air bronchograms represent types of pneumonia. However, simultaneously, it points to difficulties in differentiating them in spontaneously breathing patients. Despite this perceived difficulty, determining the type of air bronchograms should be attempted according to the POLLUS-IM guidelines because knowing the bronchogram type, static versus dynamic, helps differentiate cases of pneumonia from atelectasis when considered along with the patient’s clinical profile [2, 17]. Therefore, it is a diagnostically important feature.

The tissue sign, a Lichtenstein term, has been described in radiologist textbooks as “hepatization” of the lung because the lung parenchyma without aeration appears similar to the liver ultrasonographically. It is a more severe form of consolidation than a shred sign [3].

The nodule sign is characterized as a hypoechoic oval or circular consolidation with a hyperechoic far border and a pseudo-B-line extending from that distal border through the far field of the ultrasound screen [3]. Common differentials for the nodule sign include neoplasia, granulomatous pneumonia, i.e., fungal, verminous, and bacterial, and abscess, i.e., migrating foreign body or penetrating wound. Criteria for differentiating these causes can include the use of CD and a consideration for a more comprehensive LUS examination.

The final Vet BLUE subpleural consolidation type described is the wedge sign, which has features of both the shred and tissue signs. When found in the caudodorsal and perihilar regional views, where pneumonia is often absent, pulmonary infarction, i.e., pulmonary embolism or thrombo-embolism, should be suspected and placed into clinical context. According to POLLUS-IM recommendations, the use of CD and PD can help characterize the wedge sign’s vascular pattern, which was shown to be helpful in a single veterinary case report.

An advantage of using the Vet BLUE protocol over veterinary sliding protocols is the recording of concise regions and their respective findings, B-line scoring and its previously described subpleural consolidations, shred, tissue, nodule, and wedge signs, that may be tracked in patients with LUS pathology [3]. For example, bacterial bronchopneumonia/aspiration pneumonia would be expected in more advanced cases to have subpleural consolidation as shred and tissue signs in the Vet BLUE middle and cranial lung regions. The described Vet BLUE pneumonia paradigm is that B-lines are not as severe as shred signs, which is not as severe as tissue signs, and in this clinical course, the patient would be worsening. In contrast, the patient is improving to resolution by moving from tissue to shred to B-lines to dry lung or rather in the opposite direction [3]. This paradigm has been used with COVID-19 pneumonia in which the highly infectious disease may be imaged without transporting through a hospitalization environment, limiting COVID-19 exposure [22]. As in people, pneumonia in veterinary patients may be similarly tracked rapidly at point-of-care without the delay, transport, expense, and radiation exposure of radiography and computed tomography. A Vet BLUE tree algorithm has been published and may be used for differential diagnoses based on Vet BLUE signs and their distribution [3]. None of the guidelines for human medicine include any information on the typical location of consolidations based on their etiology. The only statement is that two-thirds of lung infarctions are located in the posterior part of the lower lung fields in humans [2, 18].

On the technical aspect, one study [23] compared the accuracy of microconvex, linear, and phased-array (PA) probes in various pulmonary abnormalities in a population of 200 animals (116 dogs and 84 cats) with dyspnea. The goals of the study were to evaluate the agreement between these three transducer types in imaging of B-lines in dogs and cats with CPE, pneumonia, and lung neoplasm and to determine the accuracy of LUS in distinguishing between CPE, pneumonia, and lung neoplasms via a LUS score designed by the authors. LUS was performed according to the Vet BLUE protocol, which included the definitions of consolidations used in this protocol. The authors concluded that the highest agreement of LUS was observed between the microconvex and linear transducer, with significant discrepancies between these two probes and the PA probe, both in the aspect of B-lines numbers and their location. Therefore, the authors do not recommend the PA probe for performing LUS in animals. The authors state that the microconvex and linear probes can be used interchangeably.

The same criteria for Vet BLUE subpleural consolidations have been studied by several authors using Vet BLUE [7, 9, 10, 16], sliding protocols [11, 12] and less specified LUS examinations, which are unclear in their methods [6, 8, 14, 15]. While the recommendations from human medicine clearly state that the largest lung surface should be examined [2, 17], the clinical status as stable or unstable must be considered, and a choice must be made between an abbreviated versus comprehensive LUS examination for the patient’s safety. Nonetheless, guidelines for people recommend that a comprehensive LUS should always be attempted [2, 17].

Vet BLUE^®^ protocol has been used to assess pulmonary contusions [13], determine the cause of cough [16], or assess the accuracy of this protocol in various thoracic pathologies [7]. Although pulmonary contusions are often detected by the presence of B-lines, subpleural consolidations are possible in more severe cases. In one study [13], Vet BLUE had a sensitivity (Se) and specificity (Sp) of 90.5% and 87.5%, respectively, for the diagnosis of pulmonary contusions using computed tomography as the reference standard, outperforming 3-view thoracic radiography (TXR), having a similar Sp but less than ideal Se of 66.7% [13]. Another protocol, named Vet-ABCDE, has also been applied to detect pulmonary contusions, finding comparable Sp and Se as Vet BLUE, again with LUS outperforming TXR [12]. However, this study did not compare to computed tomography, and the investigator was not blinded to other clinical data, including TXR findings, before recording their sliding LUS findings within the medical record versus Vet BLUE data sheets filled out immediately after the Vet BLUE study before looking at other imaging. This supports the hypothesis that LUS is an accurate tool in specific clinical situations, regardless of the protocol [13].

Another study [16] applied the Vet BLUE protocol to 100 stable dogs presenting with cough without dyspnea. The study defined pneumonia by the finding of subpleural consolidations of shred sign in gravity-dependent Vet BLUE regions of middle and cranial lung regions (Se and Sp, 57% and 92%; *N* = 7) and recorded other Vet BLUE subpleural consolidations, including the Vet BLUE nodule sign supporting the presence of metastatic neoplasia (100% and 95%, *N* = 4) [16]. Of the twenty dogs with subpleural consolidation, 11 had shred signs, 1 had a tissue sign, 9 had nodule signs, and 1 had both shred and nodule signs [16]. The final diagnosis was based on the patient’s complete clinical profile with no standard for what additional imaging was used, and 19/100 dogs had no final diagnosis, a study limitation in addition to small numbers of dogs in various final diagnosis categories.

A group of researchers has published two clinical studies in non-urgent care, one applying Vet BLUE to the mitral valve and concurrent lower respiratory tract disease dogs, and the other a non-specified LUS-thoracic POCUS protocol for subpleural consolidations and pleural space disease [8, 9]. Their Vet BLUE study focused on B-line scoring using a defined criterion for a correct diagnosis of cardiogenic pulmonary edema (CPE) but also included other findings, including pleural line characterization and subpleural consolidations and their correlation with other non-CPE diseases that may be considered as CPE false-positives. The investigators, while using a 10 − 5 MHz frequency convex probe, noted differences in the pleural line being smooth versus abnormal and thus may differentiate cardiogenic pulmonary edema (smooth, thin, hyperechoic) versus other lung pathology (thickened, blurred) with subpleural consolidations [9]. The study concluded that recognizing abnormalities, i.e., thickened or blurred pleural line and subpleural consolidations, other than B-lines, is essential for an accurate interpretation in mitral valve disease dogs that may have other pulmonary diseases or complications. The authors of this study also concluded that the presence of pleural line abnormalities or subpleural consolidations on LUS is a predictor of a false-positive result of CPE in patients with MVD [9].

The second study by these authors was retrospective, and the LUS protocol was not specified; scanning planes were either transverse or longitudinal planes, and probe type varied with a convex (most commonly), sector, or linear probe [8]. The final clinical diagnosis was compared to their non-specified protocol to identify the most common features of pneumonia and neoplasia. Atelectasis was also a LUS-thoracic POCUS finding defined as a small triangular, echogenic structure, with or without air bronchograms, differing from POLLUS-IM recommendations that define compression and resorption atelectasis with criteria presented in Table 2. These authors considered the LUS characteristics of a subpleural consolidation with a more heterogenous echotexture within consolidations was commonly associated with neoplasia. In contrast, the lack of nodules or mass lesions with the presence of a thickened or irregular pleural line or a consolidation defined as a hypoechogenic tissue-like area with an ill-defined far margin was associated with the diagnosis of pneumonia. B-lines were the most often detected abnormality (88% of cases), being at the same time the least specific finding for the diagnosis(8), but other ultrasonographic features were helpful in differentiating animals with neoplastic and inflammatory lung diseases. The study’s limitations are the small population of 65 animals (dogs and cats) and the lack of a final diagnosis of 10 animals that were excluded from the study. This study serves as a foundation for a larger, prospective study [8].

Another study [7] prospectively assessed the Vet BLUE protocol’s diagnostic accuracy for the detection of pleural fluid, pneumothorax, and lung pathology in dogs and cats [7]. In the study population, 131 patients had the Vet BLUE examination, and out of these, 31 underwent thoracic computed tomography (CT) to verify the ultrasound diagnosis [7]. The authors admit that this small sample size is one of the study’s limitations. In the subgroup of 31 patients that underwent both Vet BLUE and CT, the subpleural consolidations were reported in LUS of 4/31 patients, whereas on CT, 7/31 patients had evidence of consolidations [7]. The overall sensitivity and specificity of Vet BLUE for the diagnosis of consolidations was 58.3% and 95.6%, respectively [7]. In some sites where consolidations were found on CT, there were B-lines on Vet BLUE [7]. The authors did not attempt to diagnose lung pathologies based on LUS abnormalities, focusing on comparing LUS findings with lesions on CT using CT as the “gold standard.” They conclude that Vet BLUE holds promise as a method of detecting pulmonary consolidations that requires conformation with advanced imaging. They also conclude that Vet BLUE is designed as a regional protocol with 4 views per hemithorax and thus may miss focal disease in regions outside those views. Substituting Vet BLUE with a sliding lung technique may be preferred by covering a larger lung surface area. However, such a comparison needs more study to draw accurate conclusions.

Other authors have studied Vet BLUE to TXR and T-CT for the detection of nodular disease and, more specifically, metastatic disease. The study population was 62 dogs, and a final diagnosis of metastatic lung disease was determined in 37(60%) [10]. In contrast, pulmonary nodules were identified on CT in 25(40%) dogs [10]. In this subpopulation of 25 patients, pulmonary nodules were identified on TXR in 16/25(64%) dogs, and on Vet BLUE^®^ in 15/25(60%) of dogs [10]. TXR identified pulmonary nodules in 10/37(27%) dogs that did not have pulmonary nodules on CT, while on Vet BLUE^®^, 13/37(35%) dogs had pulmonary nodules despite not having them on CT [10]. The study found that overall TXR Se and Sp was 64% and 73%, and Vet BLUE was 60% and 65%. However, the study had a weak methodology because the order and intervals between comparative imaging modalities were not standardized. Vet BLUE’s performance may also have been confounded by performing the examination following sedation and anesthesia for CT, leading to difficulties in interpreting Vet BLUE findings due to various degrees of confounding lung atelectasis. Of note, the Vet BLUE protocol screens 24 intercostal spaces and is generally performed in a single longitudinal scanning plane. and this imaging may not perform as well as a sliding protocol of all intercostal spaces.

In a study of 66 dyspneic dogs [6], the authors used an unspecified LUS protocol similar to the regional approach of Vet BLUE to differentiate pulmonary neoplasia from pneumonia [6]. Inclusion criteria were the presence of dyspnea (> 30 breaths/min with subjective exercise intolerance). Dogs were initially evaluated by echocardiography to exclude morphological and functional abnormalities in the heart, pericardial fluid, and inflammatory or neoplastic changes in the heart [6]. The dogs then underwent a LUS examination and were included if one of the following abnormalities was found: thickened pleural line or other pleural line abnormalities; B-lines, any subpleural consolidations; a translobar consolidation, namely the tissue sign or “hepatization” of the lung; hypoechoic pulmonary nodules (diameter < 3 cm) or masses (> 3 cm in diameter) of round or oval shape with a clear-cut deep margin, grouped under one category of “tumor”; or free pleural fluid [6]. Both consolidations and hepatization may or may not have aeration, a feature corresponding to bronchograms (dynamic or static). The study group was divided according to the diagnosis into the neoplasia group (confirmed by either TXR or thoracic CT and a biopsy or thoracocentesis) and bacterial pneumonia group (confirmed by a 2-week course of a wide-range antibiotic treatment followed by LUS; if resolution of abnormalities was noted on ultrasound, the treatment was discontinued; otherwise, the treatment was continued for another 7 days and LUS was repeated) [6]. Statistical analysis was performed, and a decision tree was created. The algorithm was based on 3 of 6 LUS abnormalities on initial examination that included the presence of tumor, hepatization, and less than three versus at least three quadrants with subpleural consolidations [6]. Limitations of the study include a diagnosis of pneumonia based on response to a 2-week course of antibiotics, small patient numbers, bronchograms were not subdivided, excluding the possibility of confounding atelectasis, oversimplification of LUS abnormalities, and moderate or weak precision of statistical estimates.

Aspiration pneumonia (AP) is a disease in dogs with lesions resembling those of community-acquired pneumonia in people. One published study [11] used a sliding protocol to evaluate aspiration pneumonia in 17 dogs with AP, combining LUS findings with TXR, C-reactive protein (CRP) levels, and clinical abnormalities [11]. This prospective observational study included 17 dogs, but only six were available for the second recheck visit a month later. The study results show that the resolution of LUS abnormalities and CRP levels most closely follow the clinical picture of aspiration pneumonia [11]. The study strengths are a detailed description of consolidations, including air and fluid bronchograms; the creation of a scoring system allowing the comparison of TXR and LUS finding and their severity; and the re-evaluation of patients following treatment [11].

Yet another study [15] prospectively evaluated 68 patients (50 dogs and 18 cats) using a non-specified LUS protocol utilizing two probe types (linear and microconvex) and views in 2 different scanning planes (longitudinal and transverse) [15]. The conclusions were that LUS may be used as a supplementary examination of dyspneic animals, with diagnostic accuracy exceeding 88% (for anticoagulant poisoning) and reaching as high as 95,5% (for cardiogenic pulmonary edema) [15]. It is important to note that a radiologist performed both the radiographic and ultrasonographic examination, so this diagnostic accuracy may be lower if LUS is performed by less experienced clinicians or non-radiologists.

One case report [24] was published describing the utility of LUS for the differentiation of subpleural consolidations in a dog with *Angiostrongylus vasorum* infestation [24]. The examination was performed with a sliding technique in three lines, both perpendicular and parallel to the ribs on each side of the thorax [24]. The authors describe various types of vascularity within the consolidations, allowing for the differentiation of inflammatory and infarctual lesions according to the guidelines from human medicine. The study is the first description of the usefulness of Doppler analysis of consolidations to provide additional information on the etiology of these consolidations.

One published study [14] described a case series of 10 cats with lung lobe torsions using an unspecified LUS protocol and other imaging modalities and diagnostics, including TXR, bronchoscopy, or a combination of these modalities. The study concluded that pleural effusion was a major feature of lung lobe torsion in cats. The study did not present any specific descriptions of lung consolidations [14]. A second study [25] describes the application of CEUS for diagnosing lung lobe torsions [25]. The suspected portion of the lung was examined with both a microconvex and a linear probe, and the evaluation of lesions in CD mode was attempted but was inconclusive due to motion artifacts. Motion artifacts are a significant problem hindering CD analysis use in LUS, especially in dyspneic patients. The authors performed CEUS and found the absence or reduction of contrast enhancement in torsed portions of the lung and concluded that this finding might increase the index of suspicion for lung lobe torsions [25]. Another group of researchers published a study [26] presenting a multicenter case series describing ultrasound features of lung lobe torsion in 15 dogs. Lung lobe torsion was confirmed on thoracic CT, and LUS was performed in 2D mode with a microconvex probe. The authors describe common sonographic features of lung lobe torsion consisting of pleural fluid (15/15 dogs), hypoechoic peripheral band (14/15 dogs), and central hyperechoic parenchymal emphysema (14/15 dogs) [25]. Vascular analysis in CD mode was not attempted in all cases; therefore, it was not included in the analysis. The study is valuable, as human medicine does not describe lung lobe torsions, and no comparative data is available.

The international consensus guidelines in human medicine suggest using CEUS whenever possible to obtain further information on subpleural consolidations [17]. Apart from the already mentioned study [25], only two studies on CEUS of intrathoracic mass lesions in dogs and cats have been published [27, 28]. These are impactful, as both are well described from the technical standpoint, with a low mechanical index (complying with the guidelines for human medicine) and a thorough assessment of enhancement. The conclusions from these two studies are slightly conflicting, however, as one of them states that CEUS may be a valuable tool for the evaluation of non-cardiac thoracic lesions, allowing for the precise definition of lesion edges, the presence of necrotic areas and the distribution of pulmonary vessels [28]. At the same time, the other shows that CEUS had low sensitivity and specificity for differentiating neoplastic pulmonary masses. Therefore, findings in individual clinical patients should be interpreted with caution despite the overall usefulness of CEUS for the characterization of pulmonary and mediastinal mass lesions [27]. These reports highlight the need for further studies on the use of CEUS in veterinary patients.

## Discussion

A few conclusions can be drawn after reviewing the published studies on the use of LUS for assessing lung consolidations in veterinary patients compared to the guidelines for human medicine. Firstly, the overall number of studies on subpleural consolidations is relatively low, and most of them are not utilizing the full potential of LUS (despite the fact that they are highly impactful, valuable, and informative on other aspects of lung ultrasonography than subpleural consolidations, e.g., diagnosing and monitoring CPE). However, many of these studies were published before the guidelines for human medicine. Furthermore, many discrepancies exist in the LUS technique and interpretation of artifacts and consolidations. None of the studies presented in this critical review (apart from one case report and one on CEUS in lung lobe torsions) utilized Doppler analysis of vascularity of lung consolidations, which is often crucial in distinguishing inflammatory from neoplastic lesions. Vet BLUE^®^ examination technique is mainly used; however, finding an alternative method allowing scanning of a larger surface of the lungs may increase the accuracy of LUS in specific clinical conditions. There is also no overall consensus on the technical aspects of the examination (e.g., imaging depth, settings, probe types, etc.), with many studies incorporating only a microconvex probe, which is suboptimal for the diagnosis of subpleural consolidations, as stated in human medicine guidelines [2, 17]. It is also worth mentioning that in two studies assessing the Vet BLUE^®^ protocol, the sensitivity of LUS for the detection of consolidations or nodular disease was low (58,3% and 60%, respectively), so increasing the sensitivity of the study seems an essential future development [7, 10]. Technical differences increase operator-dependent sensitivity and specificity of LUS. For these reasons, it is imperative, in the author’s opinion, to create well-established consensus guidelines for veterinary medicine on the basic principles of LUS, both as a rapid diagnostic procedure in emergency and critical care (as a POCUS examination) and an extended protocol for internal medicine, with specific emphasis on the use of a linear probe and Doppler analysis of vascularity in subpleural consolidations.

Finally, one should remember the inherent differences between humans and companion animals, such as body weight, body condition score, anterior-posterior flattening of the chest in humans as opposed to lateral flattening of the thorax in small animals, and respiratory rates. Therefore, not all conclusions and recommendations from human medicine can be directly applied to veterinary patients. The smaller body size of dogs and cats makes the linear probe more applicable in many instances than the microconvex probe, similar to the neonatal and pediatric patients in human medicine [29–32]. Various pathologic processes in the lungs are common to humans and small animals, such as the physical mechanisms creating vertical artifacts such as B-lines or the presence of bronchograms. There also is a similarity in the representation of bacterial pneumonia, atelectasis, and nodular disease of the lungs. However, some diseases (e.g., lungworms) and pathologic processes (e.g., lung lobe torsion) are not encountered in human medicine, so no imaging studies are available for comparison. This also underlines the value of veterinary-specific recommendations and guidelines to perform LUS in companion animals consistently.

## Conclusions

LUS is a relatively new and emerging modality for small animal medicine. Several LUS protocols have been studied and applied to various lung pathologies in the past decade. Several guideline/consensus/recommendation statements have been published in human medicine. Veterinary LUS may similarly benefit by developing a more standardized approach to formats, their interpretations, and recording in medical records and terminology, in addition to exploring adjunct modalities such as CD and PD analysis of consolidations and CEUS.

## Data Availability

The datasets used and/or analyzed during the current study are available from the corresponding author upon reasonable request.

## Declarations

### Prior publication

Data have not been published previously.

## Abbreviations

AP: Aspiration pneumonia
CEUS: Contrast-enhanced ultrasound
CD: Color Doppler
CPE: Cardiogenic pulmonary edema
CT: Computer tomography
LUS: Lung ultrasound
PD: Power Doppler
POCTUS: Point-of-care thoracic ultrasound
POCUS: Point-of-care ultrasound
